# Antinociception of petroleum ether fraction derived from crude methanol extract of *Melastoma malabathricum* leaves and its possible mechanisms of action in animal models

**DOI:** 10.1186/s12906-016-1478-1

**Published:** 2016-11-29

**Authors:** Z. A. Zakaria, E. S. Jaios, M. H. Omar, S. Abd. Rahman, S. S. A. Hamid, S. M. Ching, L. K. Teh, M. Z. Salleh, S. Deny, M. Taher

**Affiliations:** 1 Department of Biomedical Sciences, Faculty of Medicine and Health Sciences, Universiti Putra Malaysia (UPM), 43400 Serdang, Selangor D.E Malaysia; 2 Integrative Pharmacogenomics Institute (iPROMISE), Universiti Teknologi MARA (UiTM), Level 7, FF3 Building, 42300 Puncak Alam, Selangor D.E Malaysia; 3 Phytochemistry Unit, Herbal Medicine Research Centre, Institute for Medical Research, Jalan, Pahang 50588 Kuala Lumpur, Malaysia; 4 Department of Biomedical Sciences, Kulliyyah of Allied Health Sciences, International Islamic University Malaysia (IIUM), 25200 Kuantan, Pahang D.M Malaysia; 5 Medical Technology Division, Malaysian Nuclear Agency, 43000 Bangi, Kajang, Selangor Malaysia; 6 Department of Family Medicine, Faculty of Medicine and Health Sciences, Universiti Putra Malaysia (UPM), 43400 Serdang, Selangor D.E Malaysia; 7 Department of Chemistry, Kulliyyah of Sciences, International Islamic University Malaysia (IIUM), 25200 Kuantan, Pahang D.M Malaysia; 8 Department of Pharmaceutical Technology, Kulliyyah of Pharmacy, International Islamic University Malaysia (IIUM), 25200 Kuantan, Pahang D.M Malaysia

**Keywords:** *Melastoma malabathricum*, Crude methanol extract, Fraction, Antinociceptive activity, Mechanisms of antinociception, Non-opioid system, Vanilloid receptors, Glutamatergic system, NO-mediated/cGMP-independent pathway

## Abstract

**Background:**

*Melastoma malabathricum* L. (family Melastomaceae) has been traditionally used as remedies against various ailments including those related to pain. The methanol extract of *M. malabathricum* leaves has been proven to show antinociceptive activity. Thus, the present study aimed to determine the most effective fraction among the petroleum ether- (PEMM), ethyl acetate- (EAMM) and aqueous- (AQMM) fractions obtained through successive fractionation of crude, dried methanol extract of *M. malabathricum* (MEMM) and to elucidate the possible mechanisms of antinociception involved.

**Methods:**

The effectiveness of fractions (100, 250 and 500 mg/kg; orally) were determine using the acetic acid-induced abdominal constriction test and the most effective extract was further subjected to the hot plate- or formalin-induced paw licking-test to establish its antinociceptive profile. Further elucidation of the role of opioid and vanilloid receptors, glutamatergic system, and nitric oxide/cyclic guanosine phosphate (NO/cGMP) pathway was also performed using the appropriate nociceptive models while the phytoconstituents analyses were performed using the phytochemical screening test and, HPLC-ESI and GCMS analyses.

**Results:**

PEMM, EAMM and AQMM significantly (*p* < 0.05) attenuated acetic acid-induced nociception with the recorded EC_50_ of 119.5, 125.9 and 352.6 mg/kg. Based on the EC_50_ value, PEMM was further studied and also exerted significant (*p* < 0.05) antinociception against the hot plate- and formalin-induced paw licking-test. With regards to the mechanisms of antinociception,: i) PEMM significantly (*p* < 0.05) attenuated the nociceptive action in capsaicin- and glutamate-induced paw licking test.; ii) naloxone (5 mg/kg), a non-selective opioid antagonist, failed to significantly (*p* < 0.05) inhibit PEMM’s antinociception iii) L-arginine (a nitric oxide precursor), but not N^G^-nitro-L-arginine methyl esters (L-NAME; an inhibitor of NO synthase), methylene blue (MB; an inhibitor of cGMP), or their respective combination, significantly (*p <* 0.05) reversed the antinociception of PEMM. Phytochemical analyses revealed the presence of several antinociceptive-bearing bioactive compounds, such as triterpenes and volatile compounds like oleoamide and palmitic acid. The presence of low flavonoids, such as gallocatechin and epigallocatechin, saponins and tannins in PEMM might synergistically contribute to enhance the major compounds antinociceptive effect.

**Conclusion:**

PEMM exerted a non-opioid-mediated antinociceptive activity at the central and peripheral levels via the inhibition of vanilloid receptors and glutamatergic system, and the activation of NO-mediated/cGMP-independent pathway. Triterpenes, as well as volatile oleoamide and palmitic acid, might be responsible for the observed antinociceptive activity of PEMM.

## Background

Pain is one of the most common manifestations of many diseases afflicting millions of people worldwide [1, 2]. It is major symptom of various ailments that incessantly producing severe physical and psychological distress for many patients at the same time disrupting their quality of life [3, 4]. In such situation, analgesics drugs are useful due to these agents ability to relieve pain without producing a loss of consciousness. Currently, three major classes of drugs are used in the pharmacological therapy of pain, namely non-steroidal anti-inflammatory drugs (NSAIDs), opioids and analgesic adjuvants, which target different components of the peripheral and central nervous system [5–7]. Unfortunately, the undesirable adverse effects such as gastrointestinal damage, renal toxicity, sedation, tolerance and respiratory depression, always overshadowed those drugs effectiveness and limited their uses [8, 9]. Due to this issue, peoples living in many developing countries in particular have been turning to natural products, particularly, those derived from medicinal plants/herbal medicines, as an alternative sources of pain-relieving agents [10, 11]. The application of plant-based natural products in the treatment of various ailments have been associated with the assumption that natural products are at least safe for consumption and cheaper than synthetically-developed drugs [12, 13].

One of the plants that have been traditionally used to treat pain is *Melastoma malabathricum* L. (family Melastomaceae) [14]. Locally known to the Malay as “*Senduduk*”, *M. malabathricum* has been used in the Malay traditional medicines to treat ailments such as stomach ulcers, dysentery and diarrhoea, those associated with pain (i.e., toothache and stomachache), to accelerate wound healing, for post-natal care and prevention of scars from small pox infection, and postpartum remedy [15–19]. Scientifically, the leaves of *M. malabathricum* have been reported to exert no acute toxicity [20] and, antibacterial [20, 21], antiviral [20], antioxidant [22], cytotoxic [22], anti-inflammatory [23, 24], anticoagulant [25], antiulcer [26], antidiarrheal [20], antinociceptive [16, 24] and antipyretic [24] activities.

In order to justify the present antinociceptive study, it is important to highlight on the previous reports related to the antinociceptive activity of *M. malabathricum*. In the two previous reports, the aqueous [24] and ethanol [16] extracts of the leaves were used and only the role of opioid receptors in modulating those extracts antinociceptive activity was investigated. In addition, we have recently published on the central and peripheral antinociceptive potential of methanol extract of *M. malabathricum* (MEMM), and reported on the involvement of vanilloid receptors, glutamatergic system and NO-mediated/cGMP-independent pathway, but not opioid receptors, in the modulation of MEMM antinociceptive activity [27]. Taking these facts into account, the present study was designed to determine the antinociceptive potential of several fractions derived from MEMM, namely petroleum ether- (PEMM), ethyl acetate- (EAMM,) and aqueous- (AQMM) extract, and to determine the mechanisms of antinociception exhibited by the most effective partition using various animal models. Briefly, the fractions were screened using the acetic acid-induced abdominal constriction test to select the most potent fraction. The most potent fraction (in this case PEMM) were then tested against the hot plate- and formalin-induced paw licking-test to establish its antinociceptive profile and subjected to further investigation on the possible mechanisms of action involving the role of opioid and vanilloid receptors, glutamate system and nitric oxide/cyclic guanosine phosphate (NO/cGMP) pathway.

## Methods

### Plant collection

The leaves of *M. malabathricum*, collected and certified by a botanist from the Institute of Bioscience (IBS), Universiti Putra Malaysia (UPM), Serdang, Selangor, Malaysia. A voucher specimen (ACP0017) has been deposited at the Herbarium of the Laboratory of Natural Products, IBS, UPM, Malaysia.

### Preparation of methanol extract of *Melastoma malabathricum* (MEMM) leaves

This procedure was performed as described in detailed by Zakaria et al. [27]. Briefly, 500 g of matured leaves that have been air-dried for 1–2 weeks at room temperature (27 *±* 2 *°*C) were grinded into powder form. The leaves were soaked in methanol in the ratio of 1:20 (w/v) for 72 h. These procedures were repeated three (3) times using the same residue. After each soaking, the supernatant was filtered using steel filter, cotton wools and Whatman no. 1 filter papers, and the supernatant collected from each extraction was pooled together. This supernatant was then subjected to evaporation process using a rotary evaporator at 40 °C under reduced pressure.

### Preparation of fractions from MEMM

The petroleum ether (PEMM), ethyl acetate (EAMM) and aqueous (AQMM) fractions were obtained from crude dried MEMM via the standard solvent partitioning methods as previously described by Sowndhararajan et al. [28]. Briefly, dried MEMM (20 g) was dissolved in 1000 mL of methanol (1:20; w/v) followed by the addition of 200 mL of distilled water. The suspension was then added with 700 mL of petroleum ether and shake thoroughly before left for 24 h to settle down into two immiscible layers wherein the lower layer (petroleum ether supernatant) was collected while the upper layer (aqueous methanol supernatant) was subjected to repeat extraction using new petroleum ether for another two times. On the other hand, the undissolved upper layer was further partitioned using ethyl acetate according to the procedures described for petroleum ether. All petroleum ether and ethyl acetate supernatants were collected and pooled together before being rotary evaporated. Each supernatant was evaporated under reduced pressure (204 Mbar) and controlled temperature (40 °C) using a vacuum rotary evaporator (Buchi Rotavapor® R210, Switzerland). In contrast, the remaining undissolved supernatant, which represents the aqueous supernatant, was collected and subjected to the freeze-drying process.

### Phytochemical screening of the most effective fraction (PEMM)

The phytochemical screening of the most effective fraction (PEMM), which was determined following the antinociceptive evaluation using the abdominal constriction test, was performed according to the standard screening tests described by Ikhiri et al. [29] but adopted by Kamisan et al. [30]. The respective test, performed to detect flavonoids, saponins, tannins, triterpenes, steroids and alkaloids, was carried out based on the 100 mg of crude extract or fractions.

### HPLC analysis of the most effective fraction (PEMM) at various wavelengths

The HPLC profile of MEMM has been published by Mamat et al. [31]. The HPLC profile of the most effective fraction (PEMM) was established according to the same methods [31]. Briefly, 10 mg of the respective fraction was suspended in 1 ml methanol and then filtered using the 0.45 μm pore size membrane filter. The filtered extract was then analysed using the HPLC system consisting of the Waters Delta 600 with 600 Controller linked to a Waters 996 photodiode array detector (Milford, MA, USA) and a 5 μm column (Phenomenex Luna; 4.6 mm i.d. × 250 mm) (Torrance, CA, USA). The sample was eluted using a solvent system consisting of 0.1% aqueous formic acid (tagged as A) and acetonitrile (tagged as B). The early condition was 95% A and 5% B with a linear gradient attaining 25% B at *t* = 12 min. This condition was retained for 10 min wherein at *t* =22 min, B was decreased to 15%, which was then retained until *t* = 30 min. The programme returned to the early solvent composition at *t* = 35 min. Throughout the analysis period, the flow rate was maintained at 1.0 ml/min while the injection volume was maintained at 10 μl. The column oven was positioned at 27 °C and the eluent was monitored at 210, 254, 280, 300, 330 and 366 nm. The retention time and UV spectra of major peaks were recorded and then analyzed. PEMM was then spiked with a list of flavonoid-based compounds (served as the standard), namely pinostrobin, hesperetin, flavanone, 4’,5,7-trihydroxy flavanone, 2,4,4’-trihydroxy chalcone, quercitrin, dihydroquercitin, fisetin, quercetin, rutin, quercitrin, naringenin, silibinin, and genistein using the same solvent system to detect their presence in the respective fraction. The HPLC analysis was carried out in the Laboratory of Phytomedicine, Medicinal Plants Division, Forest Research Institute of Malaysia (FRIM), Kepong, Malaysia.

### UHPLC-ESI Profile of the most effective fraction (PEMM)

The UHPLC system was performed on a Dionex 3000 UHPLC system acquired from Thermo Fisher Scientific (USA) that consists of an auto-sampler equipped with a column oven, a tray compartment cooler, and a binary pump with built-in solvent degasser [32]. Samples (10 μL) were injected and the chromatographic separation was performed on a BEHC18UHPLC column, 100 mm × 2.5 μm, 1.7 μm (WATERS) at a flow rate of 0.3 mL/min. The mobile phases used were (A) 0.1% formic acid in water and (B) 0.1% formic acid in acetonitrile. The separation was conducted using the following multistep gradient: initial conditions (*t* = 0 min) were 90% A and 10% B with a linear gradient reaching 15% B at *t* = 3 min. The gradient was then increased to 50% B in the next 7 min (*t* = 10 min) and further increased to 90% B for the next 2 min (*t* = 12 min). Finally, the programme was returned to the initial solvent composition at *t* = 17 min for the next analysis. The UHPLC system was coupled to a Linear Ion Trap Orbitrap mass spectrometer (Q Exactive) from Thermo Fisher Scientific (USA) equipped with an electro-spray ionization (ESI) source. The mass detection was performed in a range of 150–1500 *m/z*. The ESI source was operated in negative ion mode under the following specific conditions: source voltage: 3.2 kV; sheath gas: 35 arbitrary units; auxiliary gas: 15 arbitrary units; sweep gas: 10 arbitrary units; and capillary temperature: 320 °C. Nitrogen (>99.98%) was employed as sheath, auxiliary, and sweep gas. Instrument control and data acquisition were performed with Chameleon 6.8 software and Xcalibur 2.2 software (Thermo Fisher Scientific). The UHPLC-ESI analysis was carried out in the Laboratory of Phytochemistry Unit, Herbal Medicine Research Centre, Institute for Medical Research, Jalan Pahang, 50588 Kuala Lumpur, Malaysia.

### GC–MS analysis of MEMM and PEMM

GC-MS analysis of MEMM and PEMM were performed using the Agilent GC system (Model no. Agilent 19091S-433) attached to the gas chromatograph-interfaced to a mass spectrometer detector (GC-MSD) equipped with a HP-5MS silica capillary column (30.0 m X 250 μm X 0.25 μm nominal), composed of 5% phenyl methyl siloxane. For GC-MS detection, an electron ionization system with ionizing energy of 70 eV was used. Helium gas (99.999%) was used as the carrier gas at constant flow rate of 1 mL/min and an injection volume of 1 μL was employed (split ratio of 10:1); mode Split-Splitless Inlet; Injector temperature 250 °C (pressure 10.39 psi); Ion-source temperature 280 °C. The oven temperature was programmed from 100 °C (isothermal for 2 min) and maximum oven configuration at 325 °C, with an increase of 10 °C/min, to 200 °C, then 5 °C/min to 280 °C, ending with a 9 min isothermal at 280 °C. Mass spectra were taken at 70 eV; a scan interval of 0.5 s and fragments from 45 to 450 Da. Total GC running time was 35.50 min. The relative % amount of each component was calculated by comparing its average peak area to the total areas, software adopted to handle mass spectra and chromatograms was a Turbomass. For identification of compounds, the interpretation on mass spectrum GC-MS was conducted using the database of National Institute Standard and technology (NIST) having more than 62,000 patterns. The spectrum of the unknown component was compared with the spectrum of the known components stored in the NIST library. The name, molecular weight and structure of the components of the test materials were ascertained.

### Drugs and chemicals

The following reagents and drugs were used: methanol, petroleum ether, and ethyl acetate (Fischer Scientific, UK); dimethyl sulfoxite (DMSO), formalin, acetic acid, morphine sulphate, acetylsalicylic acid (ASA), naloxone (NLX), capsaicin, glutamate, capsazepine, L-arginine (L-arg), N^G^-nitro-L-arginine methyl esters (L-NAME) and methylene blue (MB) (Sigma, USA). The drugs were prepared by dissolving them in the saline solution. The MEMM was dissolved in the vehicle (10% DMSO) just before used. All solutions were administered in the volume of 10 mL/kg.

### Animals

Male Sprague Dawley (SD) rats (180–200 gm; 8–10 weeks old) and male ICR mice (25–30 g; 5–7 weeks old) were purchased from Che Nur Supplier, Selangor, Malaysia. The animals were kept under room temperature (27 ± 2 °C; 70–80% humidity; 12 hrs light/darkness cycle) in the Animal Holding Unit, Kulliyyah of Allied Health Sciences, International Islamic University Malaysia (IIUM), Pahang, Malaysia and were supplied with food and water ad libitum up to the beginning of the experiments. Each rats and mice were only used once. The animals were handled in accordance with the current IIUM guidelines for the care of laboratory animals and the ethical guidelines for investigations of experimental pain in conscious animals [33]. The procedures in the present study were approved by the Animal Ethics Committee of International Islamic University Malaysia [IIUM / IACUC Approval / 2016/ (9) (58)] and were performed in accordance with the Integrated Centre for Research of Animal Care and Use (ICRACU) guidelines. The number of animals and intensity of noxious stimuli used were minimum and just at the necessary amount to demonstrate the consistent effects of the treatments. In all experiments, data were collected by a blinded, randomized and controlled design. All the experiments were conducted between the 0930 and 1830 h to minimize the effects of environmental changes. All efforts were made to minimize animal suffering and to reduce the number of animals used.

### Acute toxicity study

Acute toxicity study was performed according to the guideline for testing of chemicals by adopting the OECD No. 423 procedure [34]. According to this procedure, the acute oral toxicity study was carried out using the fixed dose approaches. Rats were divided into two groups (*n* = 10) and fasted overnight. One group was then treated with a single dose of 5000 mg/kg PEMM while the other group was given vehicle (10% DMSO) (10 mL/kg) by gavage. Each animal was monitored at least once through the first 30 min after the test solutions administration, intermittently during the first 24 h and daily afterward for 14 days. Food and water were supplied ad libitum. The mortality, body weight and behavioral screening were documented daily within the 14 days observation. Any survived rats were euthanized and subjected to the macroscopic analysis. The vital organs were collected, weighted and then fixed in 10% formalin for microscopic analysis.

### Antinociceptive studies

#### Acetic acid-induced abdominal constriction test

The acetic-acid-induced abdominal constriction test was performed according to the method described by Zakaria et al. [26] with slight modification. Male ICR mice were placed in the 10 L glass beaker as observation chamber for 20 min prior to the experiment to adapt to their surroundings. The mice (*n* = 6) were pre-treated orally (*p.o*) with 10% DMSO (negative control), 100 mg/kg ASA (positive control), or PEMM, EAMM, or AQMM (100, 250, and 500 mg/kg). 60 min after the respective test solution administration, the mice were injected via intraperitoneal (*i.p*) route with 0.6% acetic acid (10 mL/kg). The animals were immediately placed individually into the observation chamber and 5 min were allowed to elapse. The abdominal constriction resulting from the injection of acetic acid consists of constrictions of the abdominal together with a stretching of at least one hind limb. The number of abdominal constrictions produced in these animals was counted cumulatively for 25 min. Antinociceptive activity, indicated by the reduction in the mean of the number of abdominal constrictions in the test groups compared to the control group, was calculated as the percentage inhibition of abdominal constrictions (percentage of inhibitory level) using the following formula: (mean of [(control - test group)/control group] X 100%).

#### Hot plate test

The hot-plate test was used to measure response latency according to the method described by Zakaria et al. [26] with some modifications. The temperature of the metal surface (Hotplate Analgesia Meter, Columbus Instruments, Model: 144-E52) was set at 50 *±* 0*.*2 °C. The time(s) elapsed between placement until the occurrence of discomfort reactions (licking paws or jumping) was recorded as the response latency time. The mice were selected a day prior to the test on the basis of their reactivity, and only those with response latencies of 5–7 s. were used in the study. The mice (*n* = 6) were pre-treated (*p.o*) with 10% DMSO (negative control), 5 mg/kg morphine (positive control or reference drug), or PEMM (100, 250, and 500 mg/kg). Sixty minutes after the respective test solution administration, the mice were placed on the heated metal surface with the arbitrary cut-off time of 20 s. This cut-off time was adopted and defined as complete analgesia as well as to avoid tissue injury. The latency to a discomfort reaction was recorded before and at 60, 90, 120, 150, 180, 210 min after the *p.o.* administration of test solutions. The prolongation of the latency times compared with the values of the controls was used for statistical comparison.

#### Formalin-Induced paw licking test

The formalin test was performed as described by Zakaria et al. [26] but with slight modifications. Pain was induced by injecting 50 μL of 5% formalin in the intraplantarly (i.pl.) into the ventral surface of the right hind paw. Rats (*n* = 6) were administered *p.o.* with 10% DMSO (negative control), 5 mg/kg morphine or 100 mg/kg ASA (both act as the positive controls), or PEMM (100, 250, and 500 mg/kg) 60 min prior to the formalin injection. Immediately after the phlogistic agent administration, the rats were individually placed into 10 L glass beaker as observation chamber. The amount of time that the animal spent licking, or biting the injected paw, considered as an indicator of pain, was recorded for the duration of 30 min in two phases, known as the early (0–5 min) and late (15–30 min) phases.

### Analysis of the possible mechanism of antinociceptive action of PEMM

#### Investigation on the role of vanilloid receptors using the capsaicin-induced paw licking test

To investigate the role of vanilloid or TRPV1 receptors in the modulation of MEMM antinociceptive action, the procedure described by Mohd Sani et al. [35] was adopted with slight modifications. Rats were pre-treated orally with 10% DMSO, capsazepine (Capz, 0.17 mmol/kg) or PEMM (100, 250, and 500 mg/kg) 60 min before capsaicin (1.6 ug/paw, 20 μL) administration via *i.pl*. route into the ventral surface of the right hind paw. Immediately after the phlogistic agent administration, the rats were individually placed in a transparent glass cage observation chamber and observed individually for 5 min. The amount of time the animals spent licking the injected paw was recorded with a chronometer and was considered as an indication of nociception.

#### Investigation on the role of glutamatergic system using the glutamate-induced paw licking test

To study the role of glutamatergic system in the modulation of PEMM antinociceptive action, the procedure described by Mohd Sani et al. [35] was performed with slight modifications. Rats were pre-treated orally with 10% DMSO, 100 mg/kg ASA or PEMM (100, 250, and 500 mg/kg) 60 min prior to glutamate injection. A volume of 20 μL of glutamate (10 μmol/paw; in normal saline) was injected via *i.pl* route into the ventral surface of the right hind paw. Immediately after the phlogistic agent administration, the rats were individually placed in a transparent glass cage observation chamber and observed individually from 0 to 15 min. The amount of time the animals spent licking or biting the injected paw was recorded with a chronometer and was considered as an indicator of nociception.

#### Investigation on the involvement of opioid receptors system using the hot plate and formalin-induced paw licking tests

To determine the role of opioid receptors in the modulation of PEMM antinociceptive activity, a separate procedure described by Mohd Sani et al. [35] was adopted with slight modifications. Four groups of animals (*n* = 6) were pre-treated (*i.p*.) with a non-selective opioid antagonist, naloxone (5 mg/kg; *i.p*.) for 15 min followed by the oral administration of 10% DMSO or PEMM (500 mg/kg). Sixty minutes later, the animals were subjected to the hot plate test and formalin test.

#### Investigation on the involvement of nitric oxide/cyclic-guanosine monophosphate (NO/cGMP) pathway using the abdominal constriction test

To determine the role of nitric oxide/cyclic-guanosine monophosphate (NO/cGMP) pathway in the modulation of PEMM antinociceptive activity, the method described by Mohd Sani et al. [35] was adopted with slight modifications. Mice (*n* = 6) were pre-treated with 20 mg/kg L-arginine, L-NAME, MB, or their respective combination (L-arginine with L-NAME or L-arginine with MB) followed 5 min later by treatment with 10% DMSO or PEMM (500 mg/kg), respectively. Sixty minutes after the administration of test solutions, the mice were injected (*i.p*) with 0.6% acetic acid.

### Statistical analysis

The data from the antinociceptive studies (*n* = 6) were expressed as means ± standard error of mean (S.E.M) and analyzed by one or two-way analysis of variance (ANOVA) followed by the respective Dunnett’s post hoc test, or Bonferroni post-test, unless otherwise stated. The different between means of treated and a control group was considered significant at *P* < 0.05. The ED_50_ (effective dose producing a 50% inhibition in relative to control value) value for all partitions following the abdominal constriction study were determined using the GraphPad Prism Software 5.0 (GraphPad Prism, USA). The calculated ED_50_ and percentages of inhibition were used to determine the most effective partition to be used in the further antinociceptive studies.

## Results

### Phytochemical screening of PEMM

The crude MEMM has been reported elsewhere to contain flavonoids, triterpenes, tannins, saponins and steroids. In comparison to MEMM, PEMM showed strong presence of triterpenes and steroids but low presence of flavonoids, tannins and saponins. No alkaloids were detected in MEMM or PEMM (Table 1).

**Table 1:** Phytochemical screening of semi-purified PEMM and comparison to crude MEMM

Class of Compounds	Samples	
	MEMM^a^	PEMM
Flavonoids	+++	+
Triterpenes	++	++
Tannins	++	+
Saponins	++	+
Steroids	+++	+++
Alkaloid	-	-

For flavonoids, tannins, triterpene and steroids - + : weak colour; ++ : mild colour; +++ : strong colour

For saponins −+: 1 – 2 cm froth; ++: 2 – 3 cm froth; +++: > 3 cm froth

For alkaloids: − +: negligible amount of precipitate; ++: weak precipitate; +++: strong precipitate

^a^Adapted from report made by Mamat et al. [31]

### HPLC profiling of PEMM at different wavelength

**Fig. 1:**
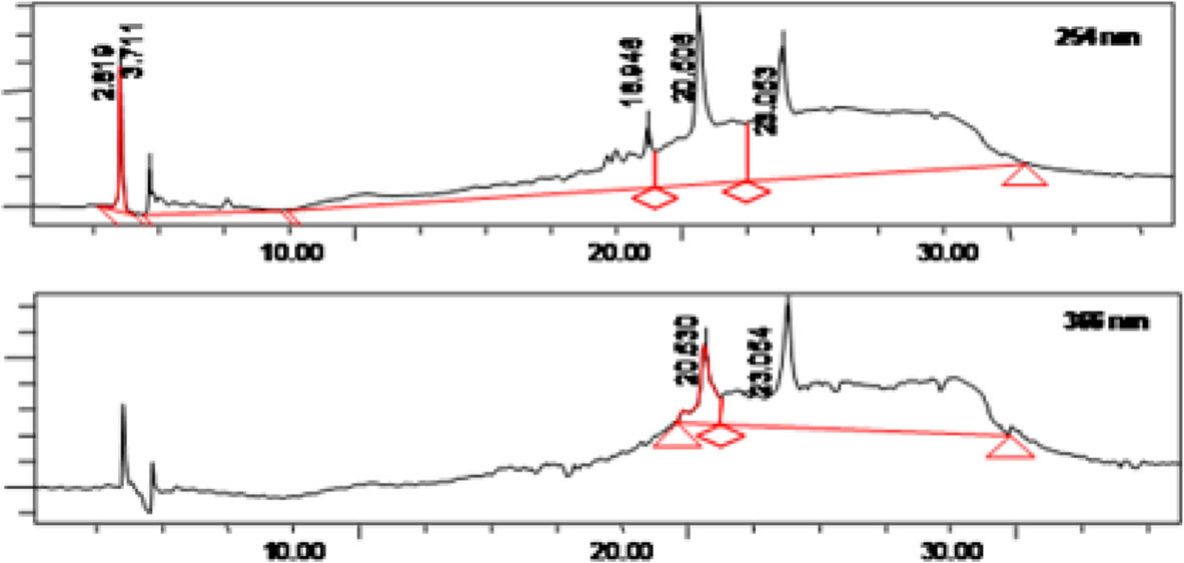
The HPLC profile of PEMM at 254 nm and 366 nm

The HPLC profile of PEMM measured at the wavelength of 254 and 366 nm is shown in Fig. 1, respectively. At 254 nm, three major peaks were detected in PEMM at the retention time (RT) of 18.946, 20.608 and 23.063 min while at 366 nm only two major peaks were detected in PEMM at the retention time (RT) of 20.630 and 23.064 min.

**Fig. 2:**
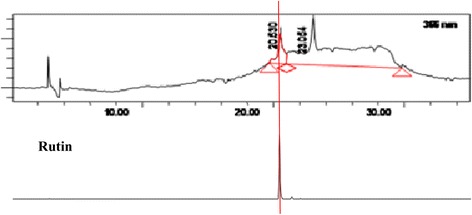
The HPLC profile of PEMM demonstrated the absence of rutin

Further analysis via spiking with standard flavonoid-based compounds available in our laboratory collection demonstrated that none of the pure flavonoid-based standards (e.g. pinostrobin, hesperetin, flavanone, 4’,5,7-trihydroxy flavanone, 2,4,4’-trihydroxy chalcone, quercitrin, dihydroquercitin, fisetin, quercetin, rutin, quercitrin, naringenin, silibinin, and genistein) were detected in PEMM. Figure 2 show that, at least, rutin’s peak, which is the closest to one of the peak of PEMM, did not perfectly match the peak with RT of 20.630 min.

### UHPLC-ESI profiling of PEMM

**Fig. 3:**
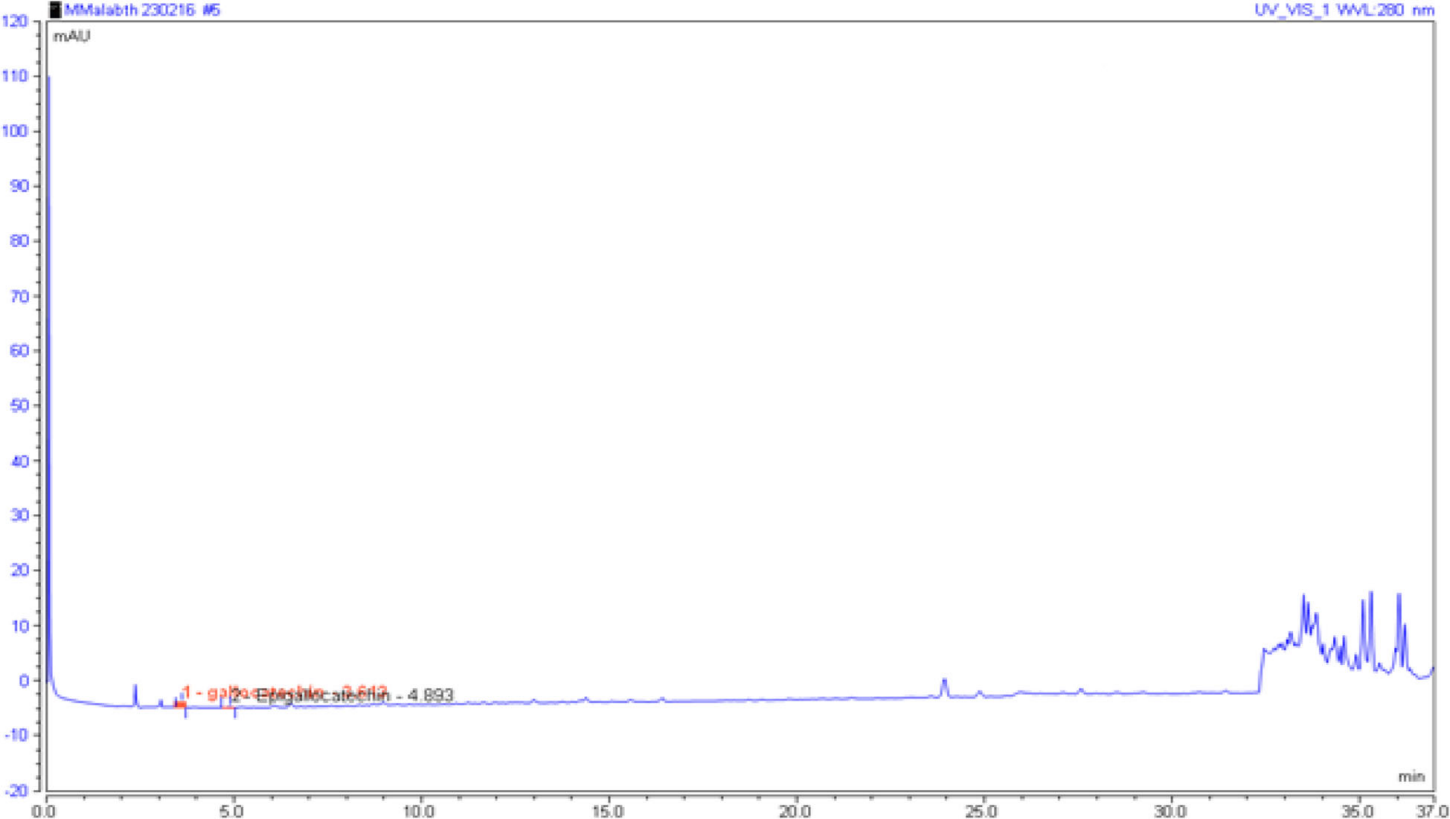
The HPLC-ESI profile of PEMM demonstrated the presence of low content of flavonoid-based compounds, namely gallocatechin and epigallocatechin

The fraction of *M. malabathricum*, PEMM, was analyzed based on the accurate mass data of the molecular ions, in which ions detected were tentatively identified by their generated molecular formula using the data analysis software (Xcalibur) that provided list of possible elemental formulas. These findings were compared together with the standard flavonoids available in the laboratory and further supported by the thorough survey of the literature. The widely accepted accuracy threshold for confirmation of elemental compositions was established at 5 ppm. The UHPLC-ESI analysis of PEMM revealed the presence of only two very small peaks indicating the presence of very small concentration of phenolic compounds, which have been identified as gallocatechin and epigallocatechin (Fig. 3).

### GC-MS profiles of MEMM and PEMM

**Fig. 4:**
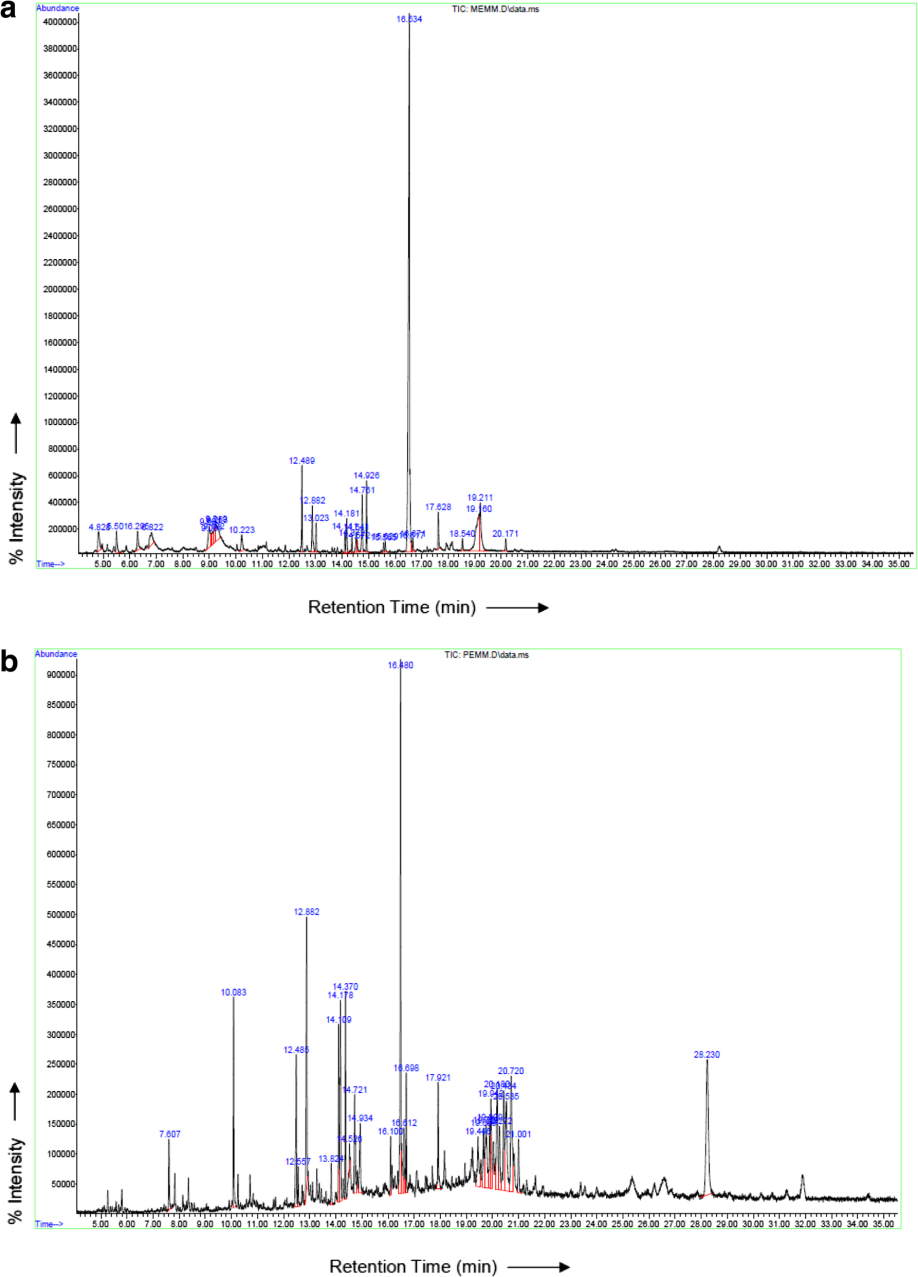
**a** The GCMS profile of MEMM. **b** The GCMS profile of PEMM

GC-MS spectra profile of crude extract (MEMM) and the most effective fraction (PEMM) are presented in the Fig. 4a and b while the identified volatile compounds are presented in Tables 2 and 3, respectively. Twenty nine volatile compounds were identified in MEMM with oleic acid amide (43.99%), 3-methylquinoline (7.84%) and propanoic acid (5.78%) being the three major volatile compounds (Table 2). Table 3 shows the volatile compounds present in PEMM wherein four major volatile compounds were detected, namely, oleic acid amide (11.54%), palmitinic acid (6.95%), methyl-25-homocholesterol (10.06%), linolenic acid methyl ester (5.69%) and phthalic acid (5.90%).

**Table 2:** GC-MS profile of the possible volatile compounds presence in MEMM

No	Retention Time (RT)	Relative Area Percentage (Area %)	Identified Compounds
1.	4.83	2.28	2-Furancarboxaldehyde
2.	5.50	1.53	α-L-Galactopyranoside
3.	6.30	1.71	2-Methyl-L-mannomethylpyranoside
4.	6.83	2.28	Pyrogallic acid
5.	9.00	2.68	α-D-Glucopyranoside
6.	9.06	0.58	Methyl β-d-Galactopyranoside
7.	9.13	1.18	β-D-Glucopyranoside
8.	9.22	1.87	β-D-Ribopyranoside
9.	9.25	2.82	Galactopyranoside
10.	10.22	1.62	Pyridine
11.	12.49	3.38	Palmitic acid
12.	12.88	2.88	Palmitinic acid
13.	13.03	1.26	Myristic acid amide
14.	14.11	0.87	Linoleic acid
15.	14.18	2.08	Linolenic acid
16.	14.37	0.56	Stearic acid methyl ester
17.	14.54	1.34	Oleic acid
18.	14.58	0.54	Pentadecanoic acid
19.	14.76	2.90	Oleic acid amide
20.	14.92	2.85	N-tetradecanoic acid amide
21.	15.56	0.40	Heptanamide
22.	15.63	0.52	Erucylamide
23.	16.54	43.99	Oleic acid amide
24.	16.63	0.56	Pentanimidic acid
25.	17.63	1.62	Dodecanedoic acid-dimetyl ester
26.	18.54	0.74	5,6-methylenedecane
27.	19.16	7.84	3-Methylquinoline
28.	19.21	5.78	Propanoic acid
29.	20.17	0.75	Squalene
Total Percentages of Identified		98.81

**Table 3:** GC-MS profile of the possible volatile compounds presence in PEMM

No	Retention Time (RT)	Relative Area Percentage (Area %)	Identified Compounds
1.	7.61	1.42	Tetradecanol
2.	10.08	3.87	Acrylic acid
3.	12.48	3.65	Palmitic acid
5.	12.88	6.95	Palmitic acid
6.	13.83	0.79	Diallylmethylsilane
7.	14.11	3.12	9,12-Octadecadienoic acid-methylester
8.	14.18	5.69	Linolenic acid methyl ester
9.	14.37	3.46	Stearic acid methyl ester
10.	14.53	0.36	Oleic acid
11.	14.72	3.01	Stearic acid
12.	14.94	2.44	1-Nonadecene
13.	16.10	1.03	Arachidic acid methyl ester
14.	16.48	11.54	Oleoamide
15.	16.62	1.53	Trifluoroacetic acid
16.	16.70	2.17	hexanedioic acid Diisooctyl adipate
17.	17.92	2.23	1,2-Benzenedicarboxylic acid
18.	20.23	2.16	2-Acetyl-N-methylaniline
19.	20.72	5.90	Phthalic acid
20.	21.00	1.69	2-methyl-Benzothiazole
21.	28.23	10.06	-Methyl-25-homochloesterol
Total Percentages of Identified		73.07	

### Acute toxicity effect of PEMM

All animals, either those that received PEMM or vehicle, exhibited a rise in body weight at weeks 1 and 2 in comparison to day 0. No changes in the behavioral pattern, mortality, or food and water intake behavior were detected among the animals were detected during the duration of experimentation. Moreover, there is no significant changes detected in the relative weight of vital organs, which was supported by the microscopic analysis that shows no signs of toxicity (data not shown). Based on these findings, PEMM was suggested to possess an LD_50_ that is greater than 5000 mg/kg body weight.

### Antinociceptive profile of fractions assessed using the abdominal constriction test

**Fig. 5:**
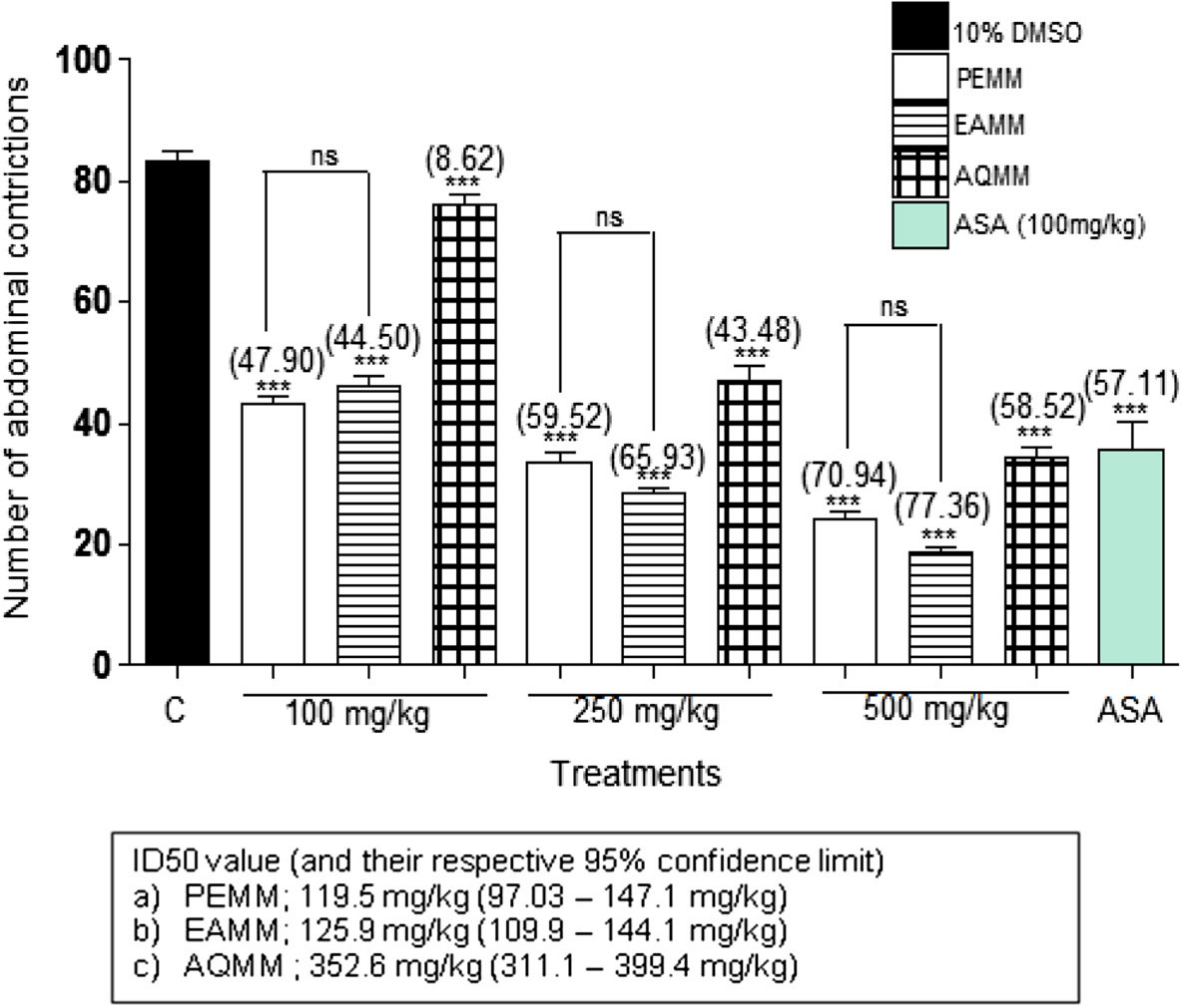
Antinociceptive activity of PEMM, EAMM and AQMM assessed using the acetic acid-induced abdominal constriction test in mice

The antinociceptive profile of PEMM, EAMM and AQMM assessed by the acetic acid-induced abdominal constriction test in mice is shown in Fig. 5. Pre-administration of PEMM, EAMM and AQMM (100, 250 and 500 mg/kg, p.o) significantly (*p* < 0.05) reduced the number of abdominal writhes induced by acetic acid in a dose-dependent manner. From the data obtained, PEMM and EAMM exerted almost similar antinociceptive efficacy but based on the calculated ED_50_ values, PEMM (ED_50_ = 119.5 mg/kg) was considered to be more effective than EAMM (ED_50_ = 125.9 mg/kg). Thus, PEMM was chosen for further antinociceptive studies.

### Antinociceptive profile of PEMM assessed using the hot-plate test

The antinociceptive profile of PEMM assessed using the hot plate test is shown in Table 4. PEMM demonstrated a significant (*p* < 0.05) antinociceptive activity in a dose-dependent manner indicated by the increase in the latency of time spend on the thermal-induced hot plate apparatus. PEMM, at 100 mg/kg, was significantly effective only at the interval of 60 and 90 min after the extract administration while, at the doses of 250 and 500 mg/kg, PEMM exerts its antinociceptive activity until the end of the experiment. Interestingly, the antinociceptive efficacy of PEMM was comparable to that of 5 mg/kg morphine (a reference antinociceptive drug).

**Table 4:** Antinociceptive activity of PEMM assessed by the hot plate test in mice

Treatment	Dose (mg/kg)	Latency of discomfort(s) at respective time interval (min)						
		0 min	60 min	90 min	120 min	150 min	180 min	210 min
10% DMSO	-	6.97 ± 0.22	6.97 ± 022	6.90 ± 0.23	6.15 ± 0.15	6.92 ± 0.23	6.88 ± 0.29	6.35 ± 0.17
Morphine	5	5.77 ± 0.15	17.37 ± 1.03^a^	18.25 ± 0.74^a^	16.52 ± 1.22^a^	13.67 ± 1.43^a^	11.22 ± 1.11^a^	10.48 ± 0.58^a^
PEMM	100	6.89 ± 0.11	9.43 ± 0.36^a^	9.67 ± 0.22^a^	7.68 ± 0.35	7.36 ± 0.20	7.51 ± 0.17	6.95 ± 0.20
	250	6.36 ± 0.27	9.60 ± 0.24^ab^	10.30 ± 0.30^ab^	10.41 ± 0.60^ab^	9.82 ± 0.44^ab^	9.22 ± 0.20^a^	7.97 ± 0.20^a^
	500	6.36 ± 0.08	11.92 ± 0.91^ab^	13.40 ± 0.48^ab^	13.38 ± 0.56^ab^	12.43 ± 0.91^a^	10.33 ± 0.27^a^	9.13 ± 0.08^a^
Naloxone (NLX)	5	6.38 ± 0.27	6.43 ± 0.41	5.98 ± 0.46	6.10 ± 0.21	5.93 ± 0.68	6.13 ± 0.58	5.67 ± 0.54
Naloxone + PEMM	5 + 500	6.71 ± 0.20	11.62 ± 0.72	13.40 ± 1.00	12.87 ± 1.00	12.00 ± 0.56	10.64 ± 0.27	8.88 ± 0.29

^a^Data differed significantly (*P <* 0.05) when compared against the control (10% DMSO-treated) group

^b^Data differed significantly (*P <* 0*.*05) when compared against the 5 mg/kg morphine-treated group

### Antinociceptive profile of PEMM assessed using the formalin-induced paw licking test

**Fig. 6:**
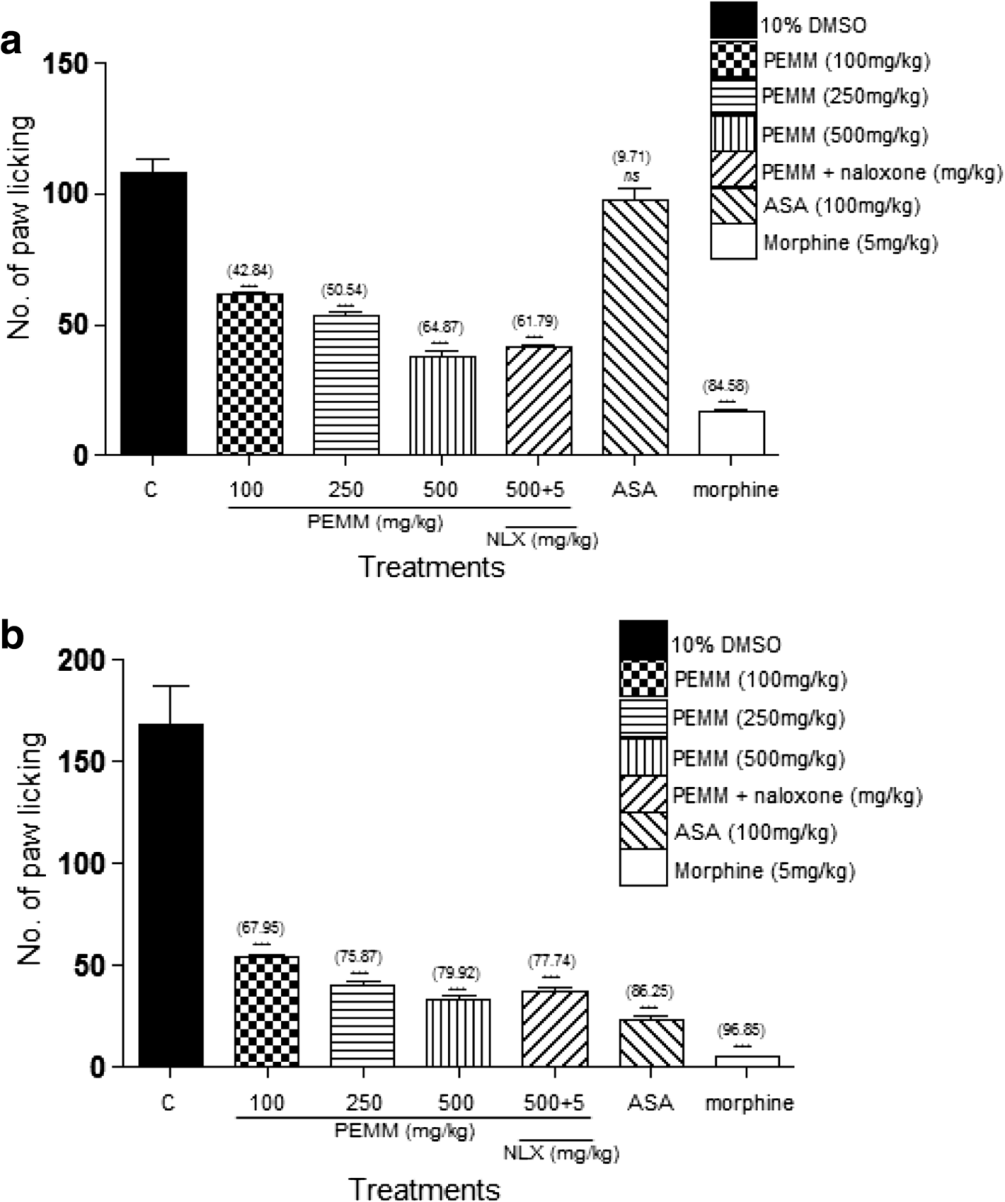
Antinocicpetive activity of PEMM assessed at the **a** early phase and **b** late phase of the formalin-induced paw licking test in rats

The antinociceptive potential of PEMM against the formalin-induced paw licking test is shown in Fig. 6a and b. Pre-treatment with PEMM caused significant (*p* < 0.05) reduction in the latency of time spends to lick the formalin-injected paw in a dose-dependent manner. Interestingly, the ability of PEMM to attenuate nociception induced by formalin can be seen in both the early and late phases, a characteristic also seen with 5 mg/kg morphine, but not 100 mg/kg ASA.

### Mechanisms of antinociceptive activity of PEMM

#### Involvement of vanilloid receptors in the antinociceptive activity of PEMM

**Fig. 7:**
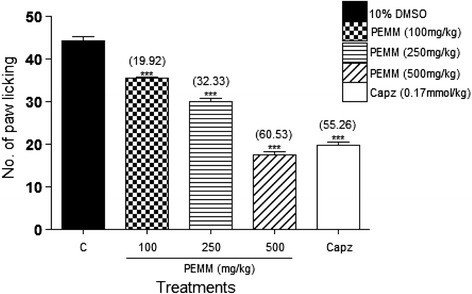
Antinociceptive activity of PEMM assessed using the capsaicin-induced paw licking test in rats

From the results obtained, PEMM, at all doses, caused a significant (*p* < 0.05) and dose-dependent inhibition of the capsaicin-induced neurogenic nociception (Fig. 7). Administration of capsazepine (vaniloid receptors’ antagonist), at 0.17 mmol/kg, significantly (*p* < 0.05) produced approximately 55.3% inhibition against capsaicin-induced nociception, which was comparable to that of 500 mg/kg PEMM (60.5%).

#### Involvement of glutamatergic system in the antinociceptive activity of PEMM

**Fig. 8:**
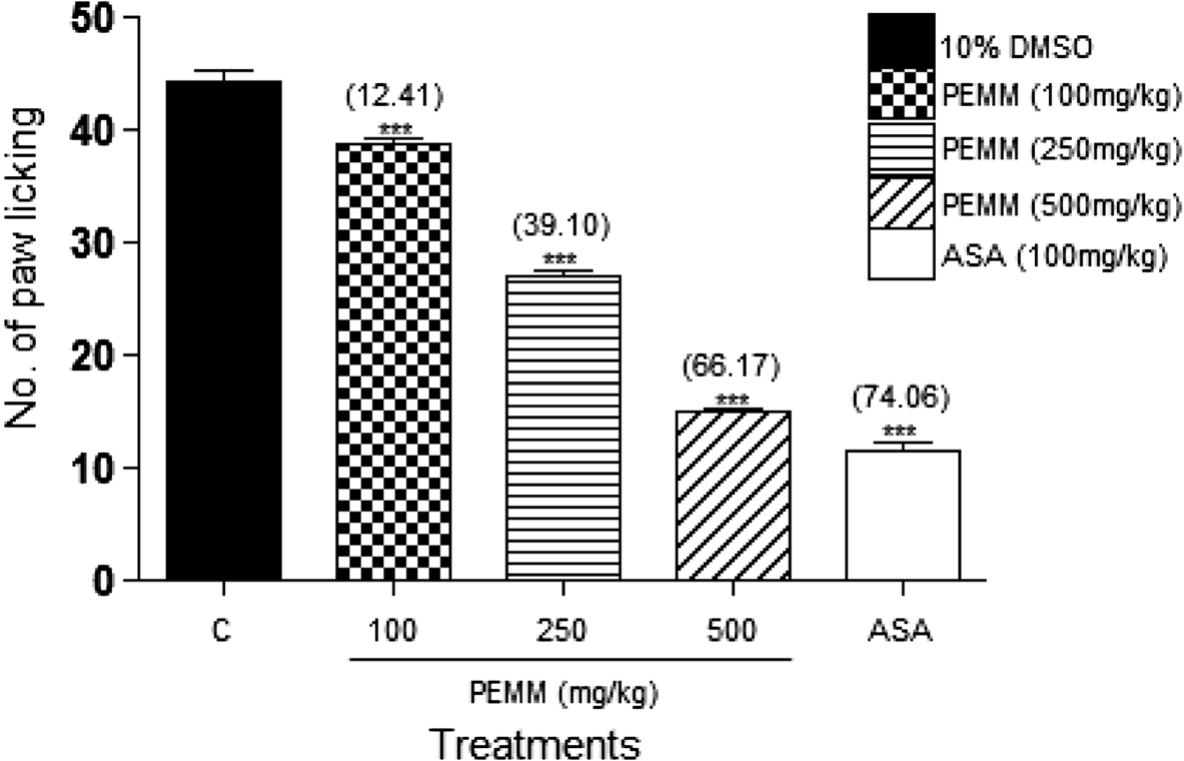
Antinociceptive activity of PEMM assessed using the glutamate-induced paw licking test in rats

From the results obtained, all doses of PEMM significantly (*p* < 0.05) reduced the latency of paw licking due to glutamate-induced nociception (Fig. 8). Administration of 100 mg/kg ASA significantly (*p* < 0.05) produced approximately 74.1% inhibition against glutamate-induced nociception, which was comparable to that of 500 mg/kg PEMM (66.2%).

#### Involvement of opioid receptors in the antinociceptive activity of PEMM

The results show that naloxone failed to significantly interfere with the antinociceptive activity of PEMM in both the hot plate and formalin-induced paw licking tests (Table 4 and, Fig. 6a and b, respectively).

#### Involvement of NO/cGMP pathway in the antinociceptive activity of PEMM

**Fig. 9:**
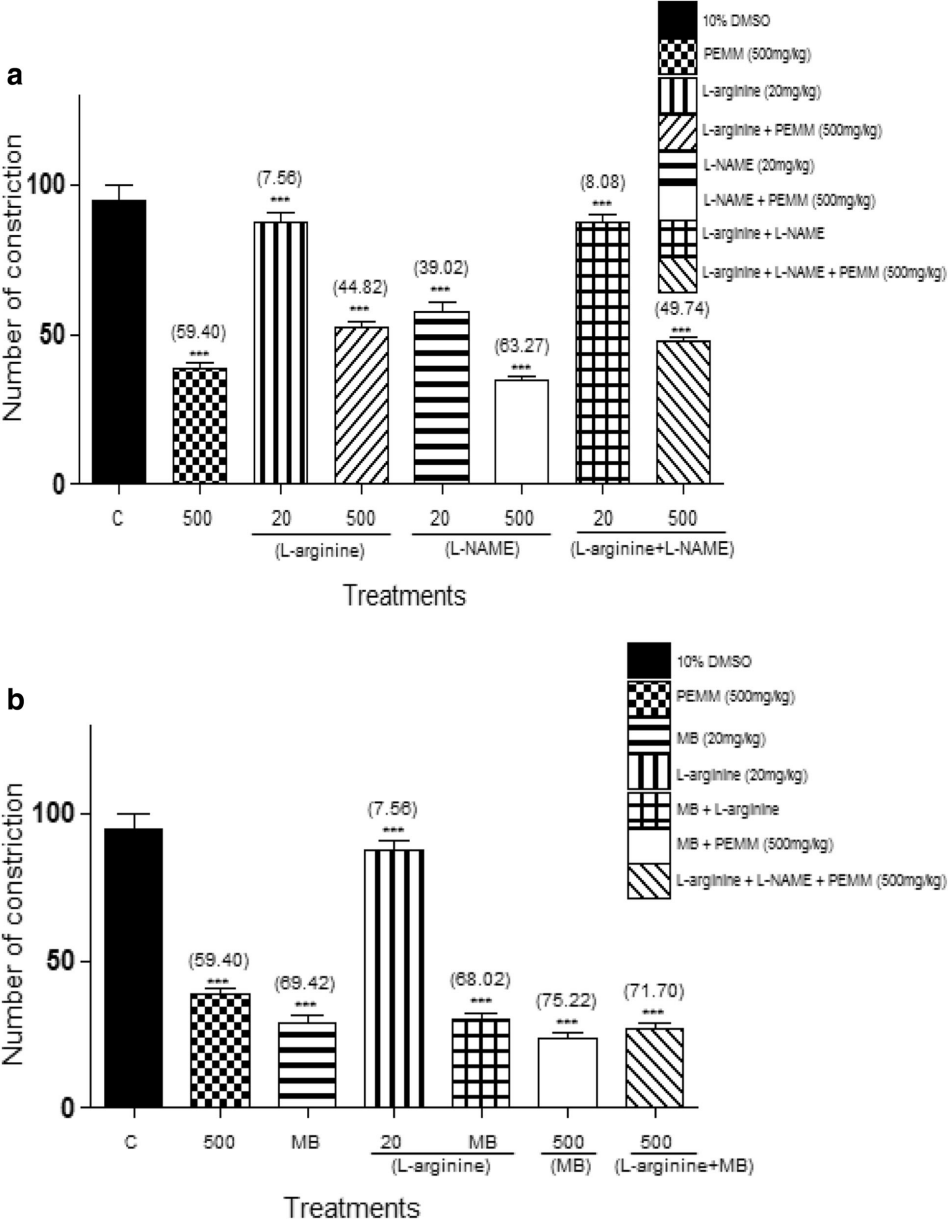
**a** Effect of L-arginine, L-NAME and their combination on PEMM antinociception as assessed by acetic acid-induced abdominal constriction test. **b**: Effect of L-arginine, MB and their combination on PEMM antinociception as assessed by acetic acid-induced abdominal constriction test

Figure 9a shows that: i) pre-treatment with 20 mg/kg L-arginine alone did not change the nociceptive intensity induced by acetic acid; ii) pre-treatment with 20 mg/kg L-NAME alone significantly (*p* < 0.05) reduced the nociceptive effect of acetic acid, and; iii) a combination of L-arginine and L-NAME (L-arginine + L-NAME) alone reversed the antinociceptive effect of L-NAME when given alone. On the other hand, pre-challenging PEMM with: i) L-arginine significantly (*p* < 0.05) reversed, but did not block, the antinociceptive activity of PEMM; ii) L-NAME did not significantly change the antinociceptive intensity of PEMM, and; iii) a combination of (L-arginine + L-NAME) significantly (*p* < 0.05) reversed, but did not inhibit, the antinociceptive activity of PEMM.

Figure 9b shows that: i) pre-treatment with MB alone induced significant (*p* < 0.05) antinociceptive activity, and; ii) pre-treatment with a combination of L-arginine and MB (L-arginine + MB) alone also demonstrated significant (*p* < 0.05) antinociceptive activity. Conversely, pre-challenging PEMM with MB or a combination of (L-arginine + MB) failed to significantly (*p* < 0.05) change the antinociceptive intensity developed following earlier pre-treatment with MB.

## Discussion

In an attempt to contribute to the discovery of alternative/new pain relieving agents with lack of unwanted side effects from medicinal plants, further antinociceptive investigations were performed on *Melastoma malabathricum* leaves. The present study was a continuation to our recently published report on the antinociceptive activity of methanol extract of *M. malabathricum* (MEMM) [27]. The justification for performing this experiment can be related to the phytoconstituents of methanol extract. Methanol is classified as a polar solvent due to the presence of hydroxyl (−OH) group. However, there is also methyl group presence in methanol, which is sort of non-polar. Due to the presence of both polar and non-polar components in methanol, compounds that dissolve in water and oils equally well including their intermediates dissolves well in methanol. According to Ahmad et al. [36], the ability to extract various types of compounds using a solvent like methanol is parallel to the ability to increase extract’s yield. Moreover, Caunii et al. [37] reported that methanol: i) can give higher concentrations of bioactive molecules from plants, and; ii) is the best solvents for extraction of different classes of phenolic compounds. These reports might be used to explain the high total phenolic content (TPC) value of MEMM [31] and supported the UHPLC analysis of MEMM, which demonstrated the presence of various flavonoids-based bioactive compounds (e.g. gallocatechin, epigallocatechin, catechin, chlorogenic acid, caffeic acid, quercetin, quercetin-3-O-glucoside, p-coumatic and hesperidin). In addition to these, the phytochemicals screening of MEMM also demonstrated the high presence of triterpenes, saponins and tannins [31]. Taking into account the presence of various classes of bioactive compounds with different polarity [27, 31], further investigations need to be carried out before the bioactive compound(s) responsible for the observed antinociceptive activity could be determined. Thus, the present study was designed to separate the bioactive compounds into non-polar, moderate polar (intermediate) and polar compounds by successive partitioning of MEMM using several solvents with different polarity to obtain the respective PEMM, EAMM and AQMM. It is worthmentioning that this attempt was performed based on suggestion made by Caunii et al. [37] that if the methanol extract expressed remarkable biological activity, further analysis towards isolation and purification of the responsible bioactive compounds should be carried out.

In the present study, the fractions (PEMM, EAMM and AQMM) were first subjected to the antinociceptive study using the abdominal constriction test to assess the antinociceptive efficacy of each extract and the results obtained show that PEMM and EAMM exerted the antinociceptive activity in almost similar intensity. However, PEMM was chosen for further antinociceptive study based on its lower ED_50_ value in comparison to the EAMM. PEMM was also found to exert antinociceptive activity against the hot plate test and both phases (early and late) of the formalin-induced paw licking test. With regards to the mechanisms of antinociception of PEMM: i) PEMM inhibited the capsaicin- and glutamate-induced paw licking test suggesting the involvement of vanilloid receptors and glutamatergic system, respectively; ii) naloxone (a non-selective opioid antagonist) failed to reverse PEMM antinociceptive activity indicating the non-involvement of opioid receptor system, and; iii) L-arginine (a nitric oxide precursor), but not L-NAME (an inhibitor of NO synthase), MB (an inhibitor of cGMP), or their respective combination, reversed the antinociceptive activity of PEMM suggesting the involvement of NO-mediated/cGMP-independent pathway.

The acetic acid-induced abdominal constriction test has been associated with the activation of peripheral nociceptive processes [38–40] and induction of acute peritoneal inflammation (localized inflammatory response) by the phlogistic agent. Moreover, the latter process occurs via the action of cyclooxygenase (COX) and increase in prostaglandins (PGE_2_ and PGF_2α_) biosynthesis [35, 40–42]. Hence, any agents capable of inhibiting the action of COX or restricting the synthesis of PGEs could be good antinociceptive agents as seen with the peripherally acting non-steroidal anti-inflamamtory drugs (NSAIDs), ASA [42, 43]. Interestingly, PEMM also attenuated the acetic-acid-induced peripheral nociception indicating the presence of analgesic principles with ability to attenuate inflammatory-mediated pain [44] by acting, partly, to inhibit the action of COX and/or synthesis of PGEs as described earlier. Unfortunately, the abdominal constriction test is considered: i) a non-specific test due to its inability to provide information on the peripheral and/or central nociceptive level inhibited by PEMM [45] and ii) to have poor specificity as it can give false positive results when use to test certain non-analgesic drugs such as muscle relaxants [46]. Thus, the applications of other nociceptive models are necessary before the final conclusion on the possible mechanisms of action adopted by PEMM could be drawn. In the present study, the hot plate test and formalin-induced paw licking test were adopted to further determine the antinociceptive activity of PEMM.

The hot plate test is selective toward the centrally-acting analgesic drugs such as opioid analgesics (such as morphine) [47] and measures the complex feedback to a non-inflammatory, acute nociceptive input resulting from a brief exposure to a noxious thermal stimulus. In the present study, PEMM successfully attenuated the thermal-induced nociceptive effect suggesting the extract ability to inhibit the central nociceptive center.

Another model of nociception, the formalin-induced paw licking test, is widely used to evaluate the ability of extracts/compounds to affect the peripheral and/or central nociceptive pathways. Being a model of persistent/continuing pain, the formalin test displays a biphasic response following the administration of formalin known as the early and late phases, which represents the respective centrally- and peripherally-mediated nociception [48]. Drugs acting at the central level (such as morphine) inhibit both phases of the formalin test in comparison to drugs acting at the peripheral level (such as ASA), which inhibit only the late phase. In the present study, PEMM was also found to inhibit both phases of the formalin-induced nociception, thus, further suggesting its ability to block the central nociceptive center. Overall, findings obtained from the three nociceptive assays implied that PEMM contains non-polar bioactive compound(s) with ability to modulate the central and peripheral nociceptive mechanisms. PEMM was also able to attenuate both the non-inflammatory- and inflammatory-mediated pain.

The roles of opioid receptors in the regulation of modulation of nociceptive processing have been demonstrated in many previous studies [49, 50]. However, the effectiveness of opioid analgesics (such as morphine) has been overshadowed by many adverse side effects (e.g. respiratory depression, vomiting, nausea, constipation, tolerance, and dependence). This is further worsening by the fact that prolongs use of morphine leads to the development of analgesic tolerance, which requires dosage increases to maintain its analgesic effect. This is problematic since dosage increases also amplify the frequency and severity of its side effects. Therefore, searching or developing new analgesics without these side effects is imperative. The non-opioid activity exerted by PEMM despite its ability to act centrally and peripherally as seen with morphine seems to be an added advantage when discussed in contact of finding new and alternative analgesics with unwanted side effects.

Furthermore, the roles of vanilloid receptors, also known as transient receptor potential cation channel subfamily V member 1 (TRPV1), and glutamatergic system in the regulation of nociceptive transmission have been reported elsewhere [51, 52]. Vanilloid receptors, are activated by capsaicin, an active ingredient in hot chili peppers, and selectively acting on neurones within the peripheral and central nervous systems [53, 54]. The present study shows that PEMM possessed antagonistic effect against the vanilloid receptors based on its ability to inhibit nociceptive transmission modulated via the vanilloid receptors. This observation was supported by earlier researches that reported on the ability of antagonists of TRPV1 receptors to exert antinociceptive activity and, to attenuate inflammatory- and neuropathic-pain [51, 52].

On the other hand, the glutamatergic system (glutamate and glutamatergic receptors) has also been acknowledged to be vital in the peripheral, spinal, and supraspinal nociceptive neurotransmission [55]. Activation of glutamatergic receptors, to a great extent, is interceded by both N-methyl-D-aspartate (NMDA) and non-NMDA receptors, and the presence or absence of NO and NO-related substances [56]. Furthermore, earlier report by Dickenson and Sullivan [57] shows that the antagonists of NMDA receptor block the spread of pain sensation and lessen the hyperexcitability of spinal cord neurons generated by C-fiber stimulation. In line with the above-mentioned reports, the present study revealed the ability of PEMM to attenuate the glutamate-induced nociceptive effect, which could possibly be achieved by acting as an NMDA receptor antagonist or by modulating the NO-mediated pathway. Interestingly, the next findings did support the latter claim that PEMM attenuated glutamate-induced nociception via the NO-mediated pathway.

The role of NO/cGMP pathway in the modulation of nociceptive transmission at the PNS and CNS levels has been well documented [58, 59]. In the earlier discussion we have highlighted that PEMM exerts a characteristic of morphine by acting at the central and peripheral nociceptive levels. Since morphine also exhibits antinociceptive activity via the NO/cGMP pathway activation [60, 61], there is a need to also evaluate the involvement of NO/cGMP pathway in the antinociceptive activity of PEMM. In the present study, PEMM antinociception was reversed by high level of NO (due to presence of L-arginine alone) but was not affected by low level of NO (due to the presence of L-NAME alone). This observation is concurrent with suggestion that the effect of NO on nociceptive response depends on dosage levels and the rate and timing of its release [58, 59]. As mentioned earlier, the presence of NO activates soluble guanylyl cyclase (sGC) leading to increase in cGMP levels, which in turn affects pain and analgesia. The role of cGMP pathway in the modulation of nociceptive process was observed when MB, an inhibitor of cGMP pathway, exerted antinociceptive activity when given alone. However, MB failed to affect the antinociceptive activity of PEMM suggesting that the antinociceptive activity of PEMM did not involved modulation of cGMP pathway. This finding also indicates that PEMM might trigger antinociceptive activity via the NO-dependent/cGMP-independent pathway. Moreover, since opioids like morphine induced antinociceptive activity via the NO/cGMP pathway, it seems reasonable to suggest that the non-opioid-acting PEMM triggered antinociceptive activity via a different pathway mediated by NO, but independent of cGMP activity (NO-mediated/cGMP-independent pathway). The role of NO-dependent/cGMP-independent pathway in the modulation of antinociceptive activity has been reported by Morioka et al. [62] and could be used to support the present observations.

Previous phytochemical screening of MEMM demonstrated the strong presence of flavonoids, triterpenes, tannins, saponins and steroids, but no alkaloids in the leaves of *M. malabathricum* [31]. On the other hand, the phytochemical screening of PEMM demonstrated the strong presence of only triterpenes with low presence of flavonoids, tannins and saponins. The low presence of flavonoids in PEMM particularly was further supported by the HPLC and UHPLC-ESI analyses. In the former analysis, only two peaks were detected at 366 nm, which upon comparison of retention time and chromatogram against 10 pure flavonoids did not match to any of them, thus, suggesting their absence in PEMM. In the latter analysis, only two small peaks were detected and identified as gallocatechin and epigallocatechin, respectively. In support of the phytochemical screenining of PEMM, Nurestri et. al. [63] isolated three pentacyclic triterpenoids, namely ursolic acid, 2-hydroxyursolic acid and asiatic acid in addition to glycerol-1,2-dilinolenyl-3-O-β-D-galactopyranoside and glycerol 1,2-dilinolenyl- 3-O-(4,6-di-O-isopropylidene)-β-D-galactopyranoside from MEMM while partitioning of MEMM using hexane (HEMM), which is also grouped as a non-polar solvent like petroleum ether, lead to the isolation of a triterpene (α-amyrin) and two amides (patriscabatrine and auranamide) [25]. Based on the presence of triterpene-based compounds as described above, further explanation with regards to the possible mechanisms of antinociception of PEMM could be plausibly suggested. For examples; i) 3β, 6β, 16β-trihydroxylup-20(29)-ene inhibited glutamate- and formalin-induced nociceptive models, and exert antinociceptive activity that is dependent on the opioid and serotonergic systems [64]; ii) siaresinolic acid reduced the acetic acid-induced nociceptive response via the activation of non-opioid system and ATP–dependent potassium channels [65]; iii) 24-hydroxytormentic acid inhibited the acetic acid- and formalin-, but not thermal-induced nociception via mechanism that did not involve modulation of the opioid, nitric oxide or serotonin systems [66], and; iv) α-amyrin and β-amyrin, in mixture, exerted significant antinociception only against the acetic acid-, capsaicin-, glutamate- and formalin-, but not thermal-induced nociceptive models in mice. Interestingly, this mixture also exhibited a non-opioid-mediated antinociceptive activity [67]. As described above, two reports [66, 67] on the failure of triterpenes to attenuate thermal-induced nocicpetion seems to contradict our findings with triterpene-rich PEMM. This discrepancy could be attributed to the low dose (30 mg/kg) of pure triterpenes used by those authors whereas in the present study PEMM fraction was effective only at the doses of 250 and 500 mg/kg.

Further analysis using the GCMS method on the presence of volatile compounds in PEMM revealed the presence of 29 volatile compounds of which five are the major compounds, namely, oleoamide (9-octadecenamide) (11.54%), 23-ethyl- (3β-23S)-cholest-5-en-3-ol (10.06%), palmitic acid (6.95%), phthalic acid (5.90%) and linolenic acid methyl ester (5.69%). Of these, at least, oleoamide and palmitic acid has been reported to show some pharmacological activities related to the antinociceptive activity of PEMM. Oleamide, for example, has been reported to exert anti-inflammatory activity when assessed using the lipopolysaccharide (LPS)-induced BV2 microglial [68]. The inhibition of inflammation occurs via inhibition of NO and PGE_2_ production. Moreover, oleamide also inhibits the activation of NFκB and PI 3-kinase, as well as the phosphorylation of inhibitor κB kinase, Akt, p38 MAPK, and ERK, and accumulation of reactive oxygen species (ROS) induced by LPS on BV2 microglial. On the other hand, Déciga-Campos et al. [69] have earlier reported on the antinociceptive activity of several palmitic acid derivatives, namely N-(4-Methoxy-2-nitrophenyl)hexadecanamide, 2-amino-3-(palmitoylamino)benzoic acid or 4-amino-3-(palmi-toylamino)benzoic acid when assessed using the abdominal constriction and hot plate tests.

## Conclusion

In conclusion, PEMM is suggested to demonstrate the non-opioid mediated antinociceptive activity at the peripheral and central level partly via the modulation of the vanilloid receptors, glutamatergic system and NO-mediated/cGMP-independent pathway. On the other hand, the antinociceptive potential of PEMM might be attributed, in part, to the presence of oleoamide and palmitic acid, the volatile compounds with pain relieving activity, as well as the higher content of triterpenes. Flavonoids, proven to be presence in low quantity in PEMM via the phytochemical screening and HPLC analyses, was further supported by the HPLC-ESI analysis that further shows the presence of low content of gallocatechin and epigallocatechin, might work synergistically with tannins and/or saponins, which also presence in low content, to further enhance the antinociceptive action of oleoamide and palmitic acid, and triterpenes.

## Abbreviations

ANOVA: One-way analysis of variance
AQMM: Aqueous partition of *M. malabathricum*
ASA: Acetylsalicylic acid
cGMP: Cyclic guanosine monophosphate
COX: Cyclo-oxygenase
DMSO: Dimethyl sulfoxide
EAMM: Ethyl acetate partition of *M. malabathricum*
EC_50_: Effective concentration that inhibits 50% of the population of sample
GCMS: Gas chromatography/mass spectrophotometry
HPLC-ESI: High performance liquid chromatography-electrospray ionization
IACUC: International Animal Care and Use Committee
L-NAME: N^G^-nitro-L-arginine methyl esters
*M. malabathricum*: *Melastoma malabathricum*
MB: Methylene blue
MEMM: Methanol extract of *M. malabathricum*
NLX: Naloxone
NO: Nitric oxide
NO/cGMP: Nitric oxide/cyclic guanosine phosphate
PEMM: Petroleum ether partition of *M. malabathricum*
TPC: Total phenolic content
TRPV1: Transient receptor potential cation channel subfamily V member 1
UPM: Universiti Putra Malaysia
